# Genome Sequence of Gordonia terrae Phage APunk

**DOI:** 10.1128/mra.01220-22

**Published:** 2023-03-06

**Authors:** Emma Anderman, Theo Dini, Kenzi Eldbah, Tara Frino, Julia Milavec, Adam Sayed, Victoria Profrock, Rachel Virtue, Theodhora Qyshkollari, Melinda Harrison

**Affiliations:** a Science Department, Cabrini University, Radnor, Pennsylvania, USA; Loyola University Chicago

## Abstract

*Gordonia* phage APunk was isolated from soil collected in Grand Rapids (MI, USA) using Gordonia terrae 3612. The genome of APunk is 59,154 bp long, has a 67.7% GC content, and contains 32 protein-coding genes. Based on its gene content similarity to actinobacteriophages, APunk is assigned to phage cluster DE4.

## ANNOUNCEMENT

Bacteriophages are ubiquitous in the environment, enormously abundant, and genetically diverse. The isolation and characterization of novel bacteriophages provide a means to better understand the evolution of viruses and their population structures, as well as their potential application in biotechnology and medicine (1). Here, we report on bacteriophage APunk, which was isolated in early September 2020 from a moist soil sample collected from a soccer field in Grand Rapids, MI (GPS coordinates, 42.915811 N, 85.644374 W) using Gordonia terrae 3612 and standard isolation procedures (2–4).

**FIG 1:**
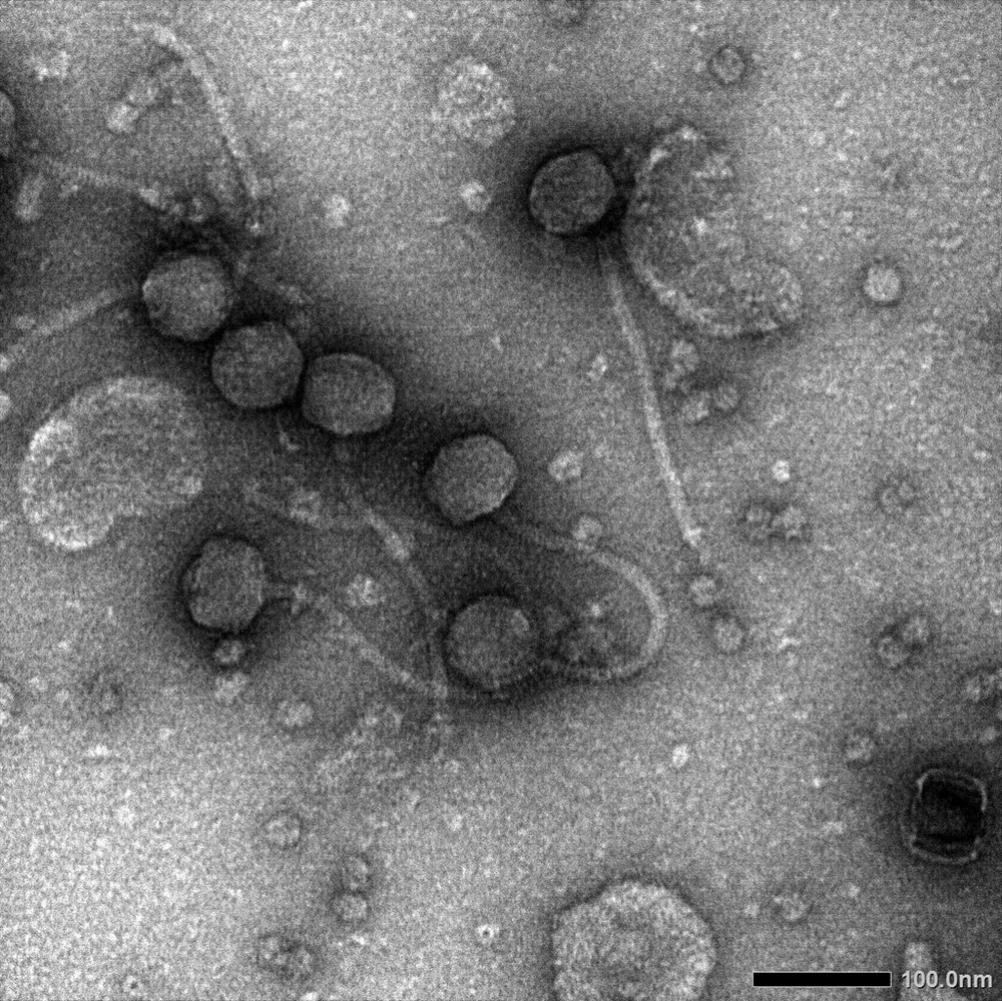
Negatively stained (1% uranyl acetate) transmission electron micrograph of phage APunk reveals a *Siphoviridae* morphology, with tails 231.7 nm (SE, 9.3 nm) long and capsids 64.4 nm (SE, 2.0 nm) in diameter (*n* = 7). The photograph was obtained from https://phagesdb.org/.

The soil sample was suspended in peptone-yeast extract-calcium (PYCa) medium; the suspension was briefly spun and the supernatant collected and filtered (pore size, 0.22 μm). The filtrate was then inoculated with Gordonia terrae 3612 and incubated with shaking at 30°C for 2 days. Following incubation, the culture was filtered; the filtrate was diluted, and the dilutions were plated in PYCa top agar with Gordonia terrae 3612. Incubation of these plates at 30°C for 1 to 2 days yielded phage APunk, which formed clear, round plaques of approximately 1 to 2 mm. APunk was purified through 3 rounds of plating. Negative-stain transmission electron microscopy revealed APunk to be a siphovirus with a tail 231.7 nm (standard error [SE], 9.3 nm) long and an icosahedral head 64.4 nm (SE, 2.0 nm) in diameter (*n* = 7) (Fig. 1).

APunk DNA lysate was isolated using the Promega DNA Wizard cleanup kit and used to prepare a sequencing library with the NEB Ultra II Library kit before sequencing on an Illumina MiSeq instrument (v3 reagents). A total of 525,256 150-base single-end reads were obtained, providing 1,256-fold coverage. The raw reads were assembled using Newbler v2.9 and checked for completeness using Consed v29.0 (5). The genome of APunk is 59,154 bp long and circularly permuted, and it has a GC content of 67.7%, which is similar to that of the isolation host, Gordonia terrae (68.4%). APunk is assigned to phage cluster DE and subcluster DE4, based on gene content similarity of at least 35% to phages in the Actinobacteriophage Database at https://phagesdb.org/ (2, 6).

The genome was annotated using DNA Master v5.23.3 (http://cobamide2.bio.pitt.edu) embedded with GeneMark v2.5p (7) and Glimmer v3.02b (8), Starterator v459 (http://phages.wustl.edu/starterator/), Phamerator v459 (https://phamerator.org) (9), BLASTp (https://blast.ncbi.nlm.nih.gov/Blast.cgi?PAGE=Proteins) (10), HHpred (https://toolkit.tuebingen.mpg.de/tools/hhpred) (11), and PECAAN v20211202, which contained the final annotation for APunk to ensure that the genome was finalized. No tRNA or transfer-messenger RNA (tmRNA) genes were detected using Aragorn v1.2.38 (12) or tRNAscan-SE v2.0 (13). All software was used with default parameters.

A total of 88 genes were identified, all of which are transcribed rightward and 33 of which could be assigned a function. These include virion structure and assembly functions, as well as holin, lysin A, and lysin B, which are encoded on the left two-thirds of the genome, and DNA metabolism functions, which are encoded on the remaining one-third of the genome. No immunity repressor or integrase functions could be identified, consistent with other cluster DE4 phages, suggesting that APunk is a lytic phage.

### Data availability.

APunk is available at GenBank under accession no. ON755186 and in the Sequence Read Archive (SRA) under accession no. SRX14443517.
